# Induction of humoral and cellular immune responses against the HIV-1 envelope protein using γ-retroviral virus-like particles

**DOI:** 10.1186/1743-422X-8-381

**Published:** 2011-08-01

**Authors:** Tea Kirkegaard, Adam Wheatley, Jesper Melchjorsen, Shervin Bahrami, Finn S Pedersen, Robert J Center, Damian FJ Purcell, Lars Ostergaard, Mogens Duch, Martin Tolstrup

**Affiliations:** 1 Department of Infectious Diseases, Aarhus University Hospital, Skejby, DK-8200 Aarhus N, Denmark; 2 SKAU Vaccines, INCUBA Science Park, Brendstrupgaardsvej, DK-8200 Aarhus N, Denmark; 3 Department of Molecular Biology, University of Aarhus, DK-8000 Aarhus, Denmark; 4 Interdisciplinary Nanoscience Center, University of Aarhus, DK-8000 Aarhus, Denmark; 5 Department of Microbiology and Immunology, University of Melbourne, Parkville 3010, VIC, Australia

**Keywords:** HIV-1 envelope protein, Virus-like particles, γ-retroviruses, immunity

## Abstract

This study evaluates the immunogenicity of the HIV envelope protein (env) in mice presented either attached to γ-retroviral virus-like-particles (VLPs), associated with cell-derived microsomes or as solubilized recombinant protein (gp160). The magnitude and polyfunctionality of the cellular immune response was enhanced when delivering HIV env in the VLP or microsome form compared to recombinant gp160. Humoral responses measured by antibody titres were comparable across the groups and low levels of antibody neutralization were observed. Lastly, we identified stronger IgG2a class switching in the two particle-delivered antigen vaccinations modalities compared to recombinant gp160.

## Findings

The induction of neutralizing antibodies remains key to developing an efficient preventive HIV vaccine. The strongest evidence in support of this comes from non-human primate studies, which demonstrate that broadly neutralizing antibodies can protect from infection [1,2]. The functional HIV envelope protein (env) complex consists of three heterodimers of the surface subunit gp120 and the transmembrane subunit gp41 arranged in trimeric spikes [3]. Gp120 binds in a sequential manner to CD4 and to a co-receptor (in most cases either CXCR4 or CCR5) on the target cell. These interactions promote extensive conformational changes in both gp120 and gp41, which leads to fusion of the viral and target-cell membranes. For vaccination purposes antibodies raised to monomeric gp120 antigens primarily target the oligomeric interface, which is not exposed in the functional trimeric structure [4]. Therefore, in order to elicit neutralizing antibodies with high efficacy directed towards the native forms of env, trimeric structure, ideally in a membrane-bound form should be a crucial property of env vaccine candidates for immunization.

The production and purification of soluble recombinant env proteins having a native trimeric conformation remains challenging [5]. As an alternative, HIV env can be presented as membrane anchored trimers on virus-like particles (VLPs) [6-8]. Several studies imply that membrane-associated trimeric env can raise higher antibody titers with increased neutralization potency compared to soluble recombinant gp120 or gp160 [9,10], and a VLP-based vaccination strategy has previously elicited some strain-specific neutralizing activity in mice and macaques [11].

In order to efficiently pseudotype MLV particles with HIV env, a cytoplasmic tail-truncated HIV env is required [12,13]. This limits the retrograde trafficking of HIV env localized on the cell surface by removing endocytosis signals within the cytoplasmic tail of gp41 [14], which in turn enhances env virion incorporation [15]. Importantly, several well-characterized neutralizing antibodies are equally potent in inhibiting both native as well as C-terminal truncated env [16].

The aim of this study was to compare the immunogenicity of env antigens delivered via γ- retroviral-like particles consisting of murine leukemia virus (MLV) gag and HIV env (termed HIV env/gag+), a purified cell fraction containing microsomes and HIV env without a viral core (termed HIV env/gag-) and recombinant gp160 (uncleaved env precursor consisting of contiguous gp120 and gp41 domains) produced in H9 cells, detergent solubilized and immuno-affinity purified [17].

**Figure 1:**
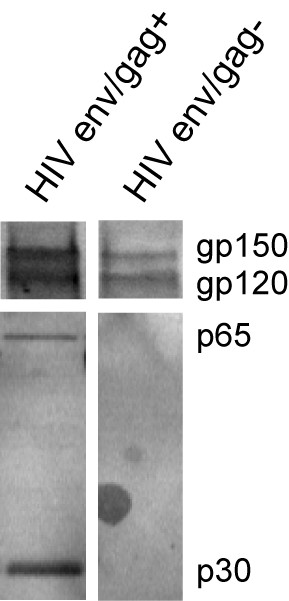
**Western blot of supernatant from transfected 293T cells pelleted through 20% sucrose using goat anti-HIV-1 gp120 (Europe Bioproducts, UK) and mouse anti-MLV CA produced by the hybridoma cell 548 (ATCC, USA)**. Primary antibodies were probed with HRP-conjugated secondary antibodies and visualized by enhanced chemoluminescence (Amersham Biosciences). Band intensities were assessed by Molecular Dynamics Storm software (GE Healthcare, DK).

We produced Moloney MLV gag particles [18] displaying env (HXB2 strain) with a truncated cytoplasmic tail (termed gp150) as previously described [13,19]. Both the VLPs and the microsome-associated HIV env were prepared by transient transfection of 293T cells followed by purification via ultracentrifugation of the supernatant through a 20% sucrose cushion [20]. Western blotting of the sedimented fractions revealed the presence of both precursor gp150 and processed gp120 in HIV env/gag+ as well as HIV env/gag- fractions (Figure 1). Equal amounts of env proteins, as determined by HIV env ELISA, were used for vaccinating mice and compared to rgp160 protein (derived from the HXB2 strain, Autogen-bioclear, UK). Six to eight week-old female BALB/c mice were handled and immunized under SPF conditions at Pipeline Biotech (Trige, Denmark) according to Danish laboratory animal legislation. Three experimental groups of five animals each received two intra-peritoneal injections 4 weeks apart with a final volume of 185 μl PBS containing 10 ug of the murine TLR9 ligand CpG oligonucleotide (ODN1826, InvivoGen) as adjuvant. The groups were denoted as either HIV env/gag+ particles, HIV env/gag- microsomes (both receiving 250 ng/injection) or soluble rgp160 (1 ug/injection, Autogen-bioclear, UK). A negative control group of three animals were immunized with PBS alone. Blood samples for pre-immune serum production were collected 5 days prior to vaccination. Mice were sacrificed four weeks after the last vaccination and blood and spleens were collected.

**Figure 2:**
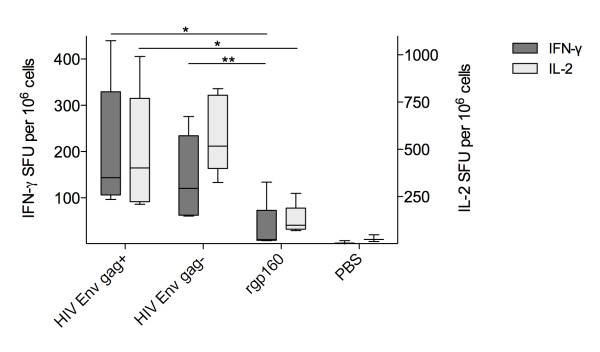
**FLUOROspot of splenocytes isolated from BALB/c mice vaccinated twice with VLPs, microsome-associated env (injecting equal amounts as determined by densitometry) or rgp160 (1 ug/injection) four weeks apart**. Box and whiskers plot with 5-95% percentile showing IFN-γ (dark) and IL-2 (light) positive counts in the three. Group medians compared with a Mann-Whitney test * p < 0.05; ** p < 0.01.

IFN-γ/IL-2 fluorospot assays (Mabtech, Sweden) were performed on splenocytes purified by ficoll-gradient centrifugation. Splenocytes from all mice were either stimulated with HIV env antigen (rgp160, 50 ng/mL) or serum-containing media alone and the number of spot forming units/10^6 ^splenocytes was determined (Figure 2). Vaccination of mice with HIV env/gag+ (median 143, range 96-439) or HIV env/gag- (median 120, range 60-276) elicited a 10-fold higher env-specific, IFN-γ^+ ^T-cell response compared with rgp160 vaccinated animals (median 10, range 7-134). For env-specific IFN-γ^+ ^secreting splenocytes there was a statistically significant increase in the HIV env/gag+ group compared to the rgp160 group (p = 0.03) whereas the increase between the HIV env/gag- vs rgp160 groups failed to reach significance (p = 0.056). Similarly, env-specific IL-2 secreting splenocytes were significantly more numerous in both HIV env/gag+ (median 401, range 209-991. p = 0.03) and HIV env/gag- (median 517, range 324-820. p = 0.008) vaccinated animals compared with animals receiving rgp160 (median 98, range 267-69) (Figure 2). There were no differences between the two particle-sized antigen groups. All statistical group comparisons were performed using a Mann Whitney test.

**Figure 3:**
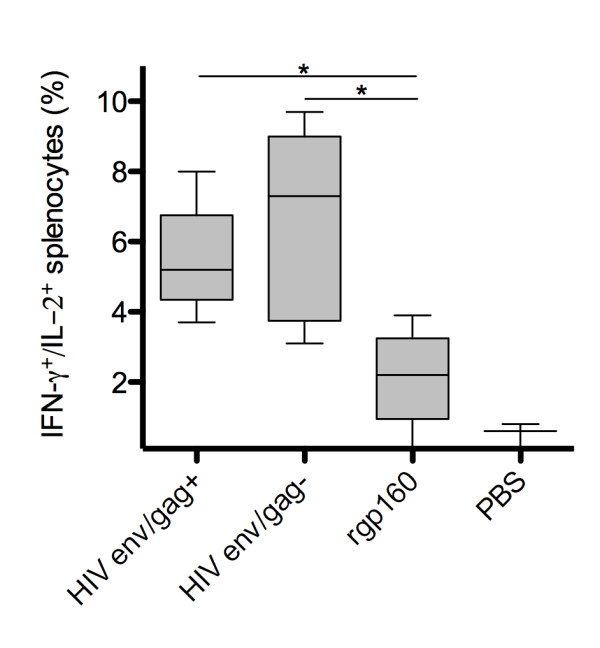
**Fraction of total antigen specific splenocytes producing both IFN-γ and IL-2**. All graphs show medians of results from five mice assayed in triplicate. Group medians were compared using the Mann-Whitney test * p < 0.05. All FLUOROspot results depicted represent antigen specific stimulation with background values (derived from unstimulated cells) subtracted.

Analyzing the proportions of env-specific cells secreting both IFN-γ^+ ^and IL-2^+ ^demonstrated higher proportions of dual positive cells in the HIV env/gag+ (5.4%, p = 0.016) and HIV env/gag- (6.5%, p = 0.015) groups compared to the rgp160 group (2.12%, Figure 3). Thus vaccination with membrane-bound HIV env immunogens both with and without a MLV viral core increased both the magnitude and the polyfunctionality of the cellular immune response compared to recombinant protein.

**Figure 4:**
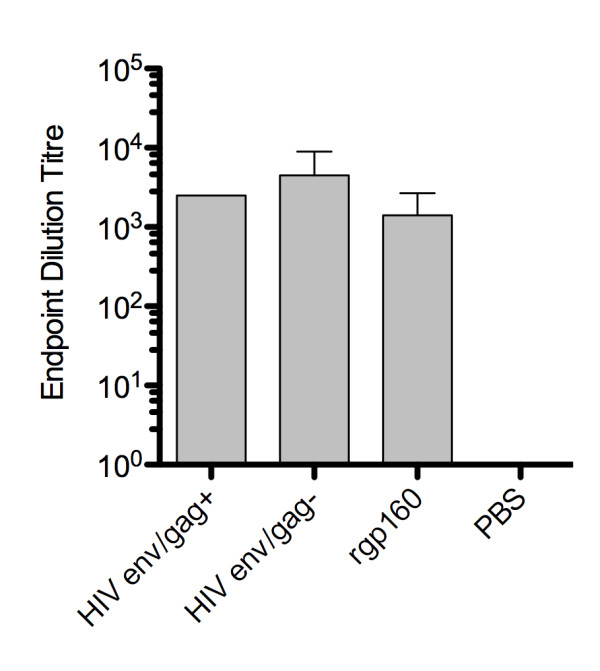
**Determination of antibody responses**. End-point dilution titre of antibodies binding rgp160. Results are average from five mice +/- SD.

The antibody response to the different HIV env immunogens was determined using two different ELISA systems. Firstly, serum IgG from vaccinated mice was analyzed for binding to rgp160. Briefly, Maxisorp Immunoplates (Nunc, Denmark) were coated with sheep anti-gp120 D7324 antibodies (Aalto Bio Reagents, Ireland) followed by incubation with 44 ng/well rgp160 (Autogen-bioclear, UK) in 5% low-fat milk PBS 0.1% Tween. Bound murine antibodies were detected using HRP-conjugated goat anti-mouse IgG (Southern Biotech, USA). The three groups had comparable anti-gp160 binding titers with mean endpoint dilutions of HIV env/gag+ 2500, HIV env/gag- 4500 and rgp160 1400 (Figure 4). A value of at least 2× the average pre-immune sera OD at each dilution from two mice was used as the cut-off for a positive value.

**Figure 5:**
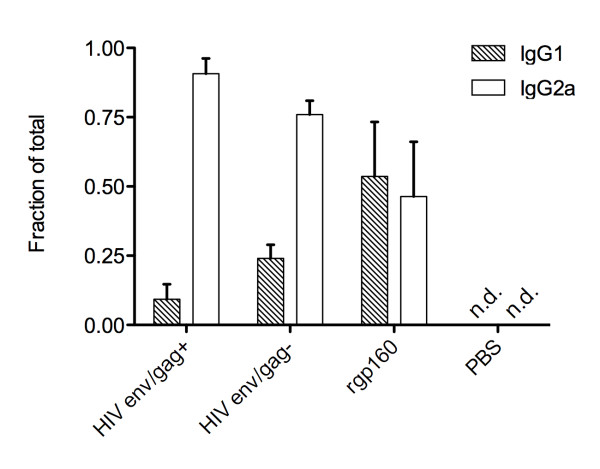
**The relative proportion of rgp160-specific IgG1 and IgG2a activity in the sera of vaccinated animals**. Average proportion from five mice in each group assayed in duplicate +/- SEM.

To further address qualitative aspects of the immune response, we determined the HIV env-specific IgG1 to IgG2a isotype ratio in the three experimental groups (Figure 5). Vaccination with either of the particle-sized antigens HIV env/gag+ or HIV env/gag- promoted class-switching of the antibody response to the IgG2a isotype. This is in contrast to vaccination with recombinant gp160 alone. Antibody class-switching to IgG2a has previously been observed in DNA plasmid-based vaccination regimens [21,22]. In the current study, we believe the presentation of membrane-associated env antigens promotes the efficient uptake and cross presentation of env to antigen presenting cells and CD4+ T cells thereby contributing to both the enhanced cellular immunity observed as well as antibody class switching.

**Figure 6:**
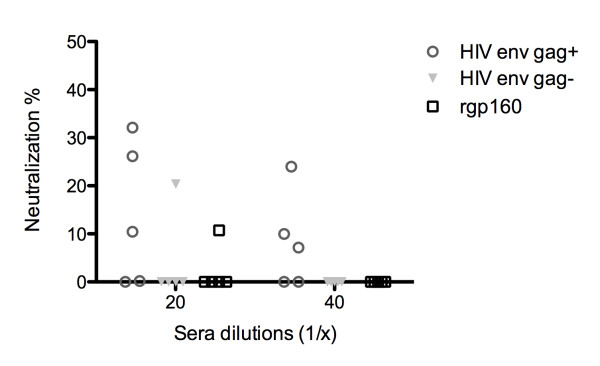
**HXB2 strain neutralization on TZM-bl cells**. Average from individual mice assayed in duplicate at indicated sera dilutions and for each mice a pre-vaccination serum sample was used to correct for individual non-specific sera inhibition.

Lastly, the ability of sera to neutralize homologous (HXB2) virus infectivity on TZM-bl cells was analyzed. As the challenge virus we utilized HXB2 virus produced in primary PBMC's. Briefly, sera from vaccinated mice and virus were incubated at indicated dilutions for 30 min at 37 C. Subsequently, virus was added to TZM-bl cells and incubated for 48 hrs upon which cells were lysed and luciferase (BriteLite, PerkinElmer, Denmark) activity was determined in a FluorStar Omega (BMG Labtech, Germany). To control for unspecific sera activity pre-vaccination sera were included for each individual mice. Neutralization was calculated as (1-[virus + immune sera/virus + pre-vaccination sera]). Generally, a low degree of neutralizing capability was observed across all three immunization groups (Figure 6). Of interest, sera from HIV env/gag+ vaccinated animals appeared to display low level of neutralization at higher sera dilutions although no statistical difference was observed.

In conclusion, these results indicate that relative to recombinant protein immunogens, γ-retroviral-based VLPs and microsome incorporated env can stimulate quantitative and qualitative improvements in T cell responses targeting HIV env. Thus, a vaccine platform using particle-delivered env trimers appears robust and immunogenic, and also holds advantages as to vector safety. The sequence similarity between γ-retroviruses and lentiviruses is very small, reducing the potential for recombination. The microsome delivery of antigen appear indistinguishable from the γ-retroviral-based VLP in terms of immunogenicity but incorporation of env was less efficient posing challenges in terms of larger scale production. Summarized, we believe these immunogenic and safety features support further investigations of particle-delivered HIV env for vaccinations against HIV.

## Competing interests

The authors declare that they have no competing interests.

## Authors' contributions

TK carried out the preparation of viral-like particles, performed the western blotting, FLUOROspot and antibody ELISA and analyzed the data. AW participated in the western blotting, the FLUOROspot and antibody ELISA. JM participated in the FLUOROspot and antibody ELISA. SB assisted in the analysis of data. FSP designed the experiments. RJC designed the experiments and helped to draft the manuscript. DFJP designed the experiments. LO coordinated the study and helped to draft the manuscript. MD conceived the study and participated in the design. MT performed the cloning, assisted in the viral-like particle preparation, designed the experiments analyzed the data and drafted the manuscript. All authors read and approved the final manuscript.
